# Practical Dietetics

**Published:** 1909-12

**Authors:** 


**Practical Dietetics.** By **W. Gilman Thompson,** Professor of Medicine in Cornell University Medical College. Fourth edition. Illustrated. Octavo, pp. 928. New York and London: D. Appleton & Co. (Cloth, $5.00.)

A book on any medical subject written by this versatile and accomplished author and teacher, is sure of a cordial reception at the hands of the medical public. The JOURNAL spoke kindly, and in praise of the three previous editions of this work and it only remains now to say this new fourth edition is better than the others.

The scientific aspect of the subject of food values, has been notably advanced during the past two years, by reason of the extensive researches of the Department of Agriculture at Washington. In order that this knowledge might become more fully available to the medical profession, Professor Thompson has completely rewritten and enlarged the present edition and added 42 new illustrations. The accepted method of dieting for each condition or disease amenable to dietetic influence has been fully and carefully considered. The tables, and summary of directions appended are helpful, as well as the discussion of the scientific principles involved.

A well regulated system of diet has much to do in the successful management of disease, not only in checking but preventing it—and a knowledge of the subject of dietetics is as important as that of materia medica. We advise all physicians who take interest in dietetics to possess themselves of this book.

